# Identification of ovarian high-grade serous carcinoma cell lines that show estrogen-sensitive growth as xenografts in immunocompromised mice

**DOI:** 10.1038/s41598-020-67533-1

**Published:** 2020-07-01

**Authors:** Alexis De Haven Brandon, Gary Box, Albert Hallsworth, William Court, Nicoll Matthews, Balint Herodek, Aitor Bermejo Arteagabeitia, Melanie Valenti, Vladimir Kirkin

**Affiliations:** Cancer Research UK Cancer Therapeutics Unit, The Institute of Cancer Research, London, SM2 5NG UK

**Keywords:** Ovarian cancer, Mechanisms of disease, Cancer models

## Abstract

Ovarian cancer remains a significant challenge in women worldwide. Tumors of the high-grade serous carcinoma (HGSC) type represent the most common form of the disease. Development of new therapies for HGSC has been hampered by a paucity of preclinical models in which new drugs could be tested for target engagement and anti-tumor efficacy. Here, we systematically assessed in vivo growth of ovarian cancer cells, including six validated HGSC cell lines, in highly immunocompromised NSG mice by varying the injection site. We found that, with the exception of OVCAR3, HGSC cell lines COV318, COV362, KURAMOCHI, OVCAR4, and OVSAHO, generally demonstrate poor growth as either subcutaneous or intraperitoneal xenografts. Intrabursal injections performed with KURAMOCHI and COV362 cells did not improve tumor growth in vivo. Additional analysis revealed that OVSAHO and COV362 express moderate levels of estrogen receptor (ERα), which translated into improved growth of xenografts in the presence of 17β-Estradiol. Surprisingly, we also found that the growth of the widely used non-HGSC ovarian cell line SKOV3 could be significantly improved by estrogen supplementation. By describing successful establishment of estrogen-sensitive HGSC xenograft models, OVSAHO and COV362, this work will enable testing of novel therapies for this aggressive form of ovarian cancer.

## Introduction

Ovarian cancer remains one of the leading causes of cancer-related deaths in women worldwide, with the all-stage 5-year relative survival rate across the globe of only 30–40%^1^. Classification of the ovarian epithelial cancers is based on histology, with the five main types being: clear cell, endometrioid, mucinous, low-grade serous, and high-grade serous tumors^2,3^. High-grade serous carcinoma (HGSC) accounts for 70% of all ovarian cancers and is also one of the more lethal forms of ovarian cancer due to its rapid growth and early spread to other organs in the peritoneal cavity^4^. Genome instability is a prominent feature in this cancer type, with aneuploidy widely reported across HGSC tumors^5^. Concomitantly, the tumor suppressor *TP53* is mutated in nearly 100% of this cancer^6–8^. Additionally, a low number of mutations are found in *NF1*, *BRCA1*, *BRCA2*, *RB1*, and *CDK12* genes. Consistently, mice with Cre-mediated deletion of *RB1* and *TP53* in the ovarian bursa develop ovarian cancer with serous histology^9^. S1

The current therapy landscape for HGSC is dominated by surgery and platinum-based chemotherapy, which has been standard of care for almost 40 years^10^. A subset of HGSC patients with *BRCA1/2* mutations is now treated with PARP inhibitors (olaparib, rucaparib, and niraparib), which were recently approved by Food and Drug Administration (FDA). Their use demonstrates the power of targeted therapy, which could significantly increase survival rates in women with ovarian cancer^11^. Development of new therapies for the HGSC has however been hampered by scarcity of easy-to-use, high-fidelity preclinical tumor models.

There is a growing number of transgenic mouse models of ovarian HGSC, based on conditional expression of oncogenes (*KRas*) or conditional deletion of relevant tumor suppressors (*Brca1/2*, *Tp53*, *Rb*, and *Pten*) in ovarian surface epithelium^9,12–16^ or fallopian tube^17–20^. However, while they are highly relevant for in vivo studies involving immunomodulation, their use also entails a number of disadvantages, including dissimilarity to the human disease, relatively long latency and high cost. On the other hand, the use of more affordable human cancer cell-line derived xenografts, with their own limitations (e.g., lack of relevant tumor-host interactions and lower predictive value), remains in the mainstream of drug development. For a long time, human ovarian cancer cell lines of a non-HGSC origin had been used as xenografts until a dedicated genetic analysis identified those with the likely HGSC genotype^21^. Consequently, two studies in 2015 investigated the growth of a panel of validated HGSC cell lines in vivo and revealed significant hurdles in establishing ovarian cancer models as either subcutaneous or intraperitoneal xenografts in immunocompromised mice. Mitra et al. found that, of 11 HGSC cell lines tested in nude mice, approximately a half (OVCAR3, OVCAR4, OVCAR5, OVCAR8, CA-OV-3, and OVSAHO) formed intraperitoneal tumors with HGSC histology, while far fewer cell lines (OVCAR3 and OVCAR5) gave rise to usable subcutaneous xenografts^22^. Elias et al. managed to grow small subcutaneous xenografts from two additional HGSC cell lines, KURAMOCHI and OVSAHO, using the highly immunodeficient NSG (NOD scid gamma) mouse strain^23^.

Ovarian cancers have been shown to express hormone receptors, progesterone receptor (PR) and estrogen receptor (ER), which correlate with the survival in some ovarian tumor entities. For example, strong PR, but not ER, expression was associated with improved survival in HGSC patients^24^. In addition, active ERα signaling and estrogen-dependent growth was described in established ovarian cancer cell lines and those derived from patients with HGSC tumors. However, the ERα expression status alone was not predictive of the response of the cell lines to the estrogen treatment^25^. In addition, expression of ER and PR in ovarian HGSC has been reported to be very variable and dependent on age^26^. Notwithstanding, the use of hormone-sensitive cell lines as xenografts, which can be used readily and reliably for modeling ovarian HGSC, has not been extensively investigated to date.

Here, we report a study in which we systematically assessed and compared the growth of HGSC (COV318, COV362, KURAMOCHI, OVCAR3, OVCAR4, and OVSAHO) and non-HGSC (IGROV1, JHOC-5, OVK18, and SKOV3) ovarian cancer cells, as well as their luciferase (Luc)-transfected variants, in highly immunocompromised female NSG mice. We describe their growth characteristics as subcutaneous, intraperitoneal, and intrabursal models and identify OVSAHO and COV362 as estrogen-sensitive subcutaneous xenograft models suitable for preclinical drug development. We also show that the very well established non-HGSC cell line SKOV3 shows sensitivity to estrogen supplementation for its growth as a tumor xenograft, suggesting a broader role of hormone signaling in modeling different types of ovarian cancer in vivo.

## Materials and methods

### Cell lines

Cell lines (Table 1) were obtained from central repositories and cultured in the specified culture medium, supplemented with 10% fetal bovine serum (FBS; unless otherwise stated), at 37 °C in 5% CO_2_. Authentication of the cells was established in-house by short tandem repeat (STR) profiling, with results being matched to online databases, e.g., https://www.dsmz.de/fp/cgi-bin/str007.cgi with an 80% acceptance rate. Cells were regularly screened for *Mycoplasma* using a PCR-based assay and were cultured for no longer than 8 weeks. Cell lines that expressed firefly luciferase (Luc) were transduced with a Luc-encoding lentiviral system. The open reading frame (ORF) encoding a firefly Luc was taken from pGL4.1 (Promega) as a *Nhe*I/*Xba*I fragment and ligated into the multiple cloning site (MCS) of pCDH-CMV-MCS2-EF1-Hygro (System Biosciences Inc). Cells were then selected using hygromycin B (Thermo Fisher 10687010), with the optimal concentration having been determined for each individual cell line from kill curves obtained previously on the parental lines. The transduction efficiency was high with these cell lines (apart from OVSAHO), so no colony selection was required, and all transduced cells were used for expansion. Cell lines were checked for luminescence on the In Vivo Imaging System (IVIS) Lumina X5 (Perkin Elmer) prior to implantation in vivo.

**Table 1:** Cell lines evaluated in the study.

Cell line	Type	Source	Medium
JHOC5	Non-HGSC	RCB (Cat No 1520)	RPMI (10% FBS)
SKOV3	Non-HGSC	ICR cell bank	RPMI (10% FBS)
IGROV1	Non-HGSC	ICR cell bank	RPMI (10% FBS)
OVK18	Non-HGSC	RCB (Cat No 1903)	RPMI (10% FBS)
COV318	HGSC	ECACC (Cat No 07071903)	DMEM (10% FBS)
COV362	HGSC	ECACC (Cat No 07071910)	DMEM (10% FBS)
KURAMOCHI	HGSC	JCRB (Cat No 0098)	RPMI (10% FBS)
OVCAR3	HGSC	ATCC (Cat No HTB-161)	RPMI (20% FBS, 10 μg/ml insulin)
OVCAR4	HGSC	ICR cell bank	RPMI (10% FBS)
OVSAHO	HGSC	JCRB (Cat No 1046)	RPMI (10% FBS)
MCF7	ER^+^ breast cancer (used as a control)	ATCC (Cat No HTB-22)	DMEM (10% FBS)

Cell line types presented are after Domcke et al.^21^.

*ATCC* American Type Culture Collection, *Cat No* catalogue number, *ECACC* European Collection of Authenticated Cell Cultures, *ER* estrogen receptor, *FBS* fetal bovine serum, *HGSC* high-grade serous carcinoma, *ICR* Institute of Cancer Research London, *JCRB* Japanese Cancer Research Resources Bank, *RCB* RIKEN cell bank.

### Protein analysis by Western blot

Approximately 2–3 × 10^6^ cells were seeded in 10-cm dishes and incubated overnight, after which the cells were rinsed with ice-cold Ca^2+^–Mg^2+^-free PBS (D8537, Sigma-Aldrich) and lysed in 500 μl ice-cold lysis buffer [150 mM NaCl, 50 mM Tris–Cl, pH 7.4, 0.5 mM EDTA, 1% Triton X-100; freshly supplemented with 1 mM NaF, 1 mM NaVO_3_, 1 mM PMSF, 1% protease cocktail (Sigma-Aldrich, Cat No P8340), 2% phosphatase inhibitors 2 (Sigma-Aldrich, Cat No P5726), and 2% phosphatase inhibitors 3 (Sigma-Aldrich, Cat No P0044)]. Lysates were gently passed three times through a 27G needle, after which they were cleared by centrifugation at 11,000*g* for 15 min at 4 °C. Supernatants containing soluble proteins were collected, and protein content was determined using the Pierce BCA Protein Assay Kit (Thermo Fisher Scientific, Cat No 23227). Proteins were resolved on a NuPAGE 4–12% Bis–Tris protein gel (Thermo Fisher Scientific, Cat No NP0332) and transferred onto Immobilon-P PVDF membrane (Merck Millipore, Cat No IPVH00010). Immobilized proteins were detected using respective primary antibodies: rabbit anti-mouse estrogen receptor 1α (1:100; Cell Signaling Technology, Cat No 13258), rabbit anti-mouse GAPDH (1:2000; Cell Signaling Technology Cat No 2118); and a secondary antibody [HRP-linked anti-rabbit IgG (1:2000; Cell Signaling Technology Cat No 7074)].

### Two-dimensional (2D) and three-dimensional (3D) cell proliferation assays

For the analysis of cell proliferation in 2D conditions, cells were seeded at 4 × 10^3^ cells/well in 96-well plates (Corning, Cat No 3603) in charcoal-absorbed media (Thermo Fisher Scientific, Cat No 32404014 and 31053028), supplemented with 10% activated charcoal-absorbed FBS (PAN, Cat No P30-2301) and, if required, 500 pg/ml 17β-Estradiol (Sigma-Aldrich, Cat No E2758), and then incubated for the indicated period of time. CellTiter-Glo (Promega, Cat No G7570) was used to assess cell proliferation as directed by the manufacturer. Alternatively, cell growth was observed using the IncuCyte S3 CMP cell imager (Essen BioScience). Cell counts were performed in triplicates.

For 3D spheroid assay, cells were seeded at 4 × 10^3^ cells/well in Ultra-Low Attachment (ULA) 96-well plates (Nexcelom Bioscience, Cat No ULA-96U-020) in charcoal-absorbed media, supplemented with 10% activated charcoal-absorbed FBS and, if required, 500 pg/ml 17β-Estradiol, and then incubated for the indicated period of time. Spheroid growth was visualized every day for 8 days, using the Celigo Imaging Cytometer (Nexcelom Bioscience), while the medium was replenished every second day. Individual spheroids from six wells per cell line per condition were analyzed.

### In vivo studies

All animal studies and breeding were approved by the institutional Animal Welfare Ethical Review Body (AWERB) and carried out in accordance with UK Home Office Regulations under the Animals (Scientific Procedures) Act 1986 and national guidelines^27^. All cell lines were tested in 6–7-week-old, female non-obese diabetic (NOD), severe combined immunodeficiency (scid), IL2 receptor common gamma chain mice, NSG (NOD.*Cg-Prkdc*^*scid*^*Il2rg*^*tm1Wjl*^/SzJ), bred at the Institute of Cancer Research (ICR) London.

For subcutaneous (SC) tumor xenograft studies, 5 × 10^6^ cells were mixed with 50% Matrigel (Corning, Cat No 354248) and implanted subcutaneously into either one or two flanks. Once visible, tumors were measured across two diameters, and volumes were calculated using the formula V = 4/3π [(d1 + d2)/4]^3^. Body weights were also recorded. Once tumors approached Home Office License limits (i.e. maximum mean diameter of 1 cm), or experiments exceeded 150 days, mice were culled and tumors were weighed and snap or viably frozen, if present.

For intraperitoneal (IP) tumor xenograft studies, 1 × 10^7^ cells were mixed with 50% Matrigel and implanted into the peritoneal cavity. Mice injected with parental cell lines were weighed and palpated weekly to assess for signs of tumor burden. Mice injected with cell lines expressing the Luc, in addition to the above methods of monitoring, were imaged weekly using the in vivo imaging system (IVIS) Lumina X5 (PerkinElmer) after an intraperitoneal injection of Luciferin (PerkinElmer, Cat No 122799) at 150 mg/kg body weight. Total flux was measured using the Living Image software.

For intra-bursal models, COV362 and KURAMOCHI cell lines, both parental and transduced with the Luc, were implanted (5 × 10^4^ cells in 5 µl) in both ovarian bursae of the isoflurane-anesthetized mice, to see if the growth rate could be enhanced. Animals were weighed and palpated weekly. Mice implanted with the Luc-expressing cells were also imaged on the IVIS once weekly as above.

For estrogen supplementation studies, mice were ovariectomized, under isoflurane anesthesia, using aseptic techniques. After 10 days, half the mice were implanted subcutaneously in the nape of neck with a 3-mm, 60-day release, 0.36-mg 17β-Estradiol pellet (Innovative Research of America, Cat No SE-121). Cell implantation and tumor monitoring were initiated 24–48 h post pellet implant.

For all studies, once a substantial weight gain was witnessed (i.e. ascitic fluid build-up), a high-flux reading was observed on IVIS, or a large mass could be palpated, mice were culled, and tumors collected for analysis.

## Results

### Growth of ovarian cancer cells as subcutaneous xenografts in NSG mice

The advent of highly immunocompromised mouse strains, such as NSG, which lack mature T cells, B cells, and NK cells^28^, allows engraftment and growth of human tumors with an unprecedented efficiency^29^. We tested a panel of ten ovarian cancer cell lines (Table 1), including the validated HGSC cells^21^, for their growth in a subcutaneous location in the female NSG mice. The results obtained with the cells inoculated in one or both flanks either as parental cell lines or those expressing the firefly Luc are summarized in Table 2.

**Table 2:** Growth of ovarian cancer cells as subcutaneous xenografts in female NSG mice.

Cell line	Type	Tumorigenic?	Days to license limit
**Parental cell lines**			
JHOC5	Non-HGSC	Yes (4/5 sites)	~ 69
SKOV3	Non-HGSC	Yes (10/10 sites)	~ 35
IGROV1	Non-HGSC	Yes (5/5 sites)	~ 34
OVK18	Non-HGSC	Yes (5/5 sites)	~ 43
COV318	HGSC	No (0/5 sites)	n.g.
COV362	HGSC	No (0/5 sites)	n.g.
KURAMOCHI	HGSC	Partially (3/5 sites)	> 105
OVCAR3	HGSC	Yes (5/5 sites)	~ 33
OVCAR4	HGSC	Yes (5/5 sites)	~ 148
OVSAHO	HGSC	Yes (10/10 sites)	~ 97
**Cells expressing luciferase (Luc**^+^**)**			
JHOC5 Luc^+^	Non-HGSC	No (0/5 sites)	n.g.
IGROV1 Luc^+^	Non-HGSC	Yes (5/5 sites)	~ 105
OVK18 Luc^+^	Non-HGSC	Yes (5/5 sites)	~ 43
COV318 Luc^+^	HGSC	No (0/5 sites)	n.g.
COV362 Luc^+^	HGSC	No (0/5 sites)	n.g.
KURAMOCHI Luc^+^	HGSC	No (0/5 sites)	n.g.
OVCAR4 Luc^+^	HGSC	No (0/5 sites)	n.g.

Cell line types presented are after Domcke et al.^21^. The term “sites” (short for “tumor implantation sites”) is defined as the number of subcutaneous cell implantation points across multiple animals.

*HGSC* high-grade serous carcinoma, *Luc*^*+*^ luciferase-expressing cells, *n.g.* no growth, *NSG* NOD scid gamma.

**Figure 1:**
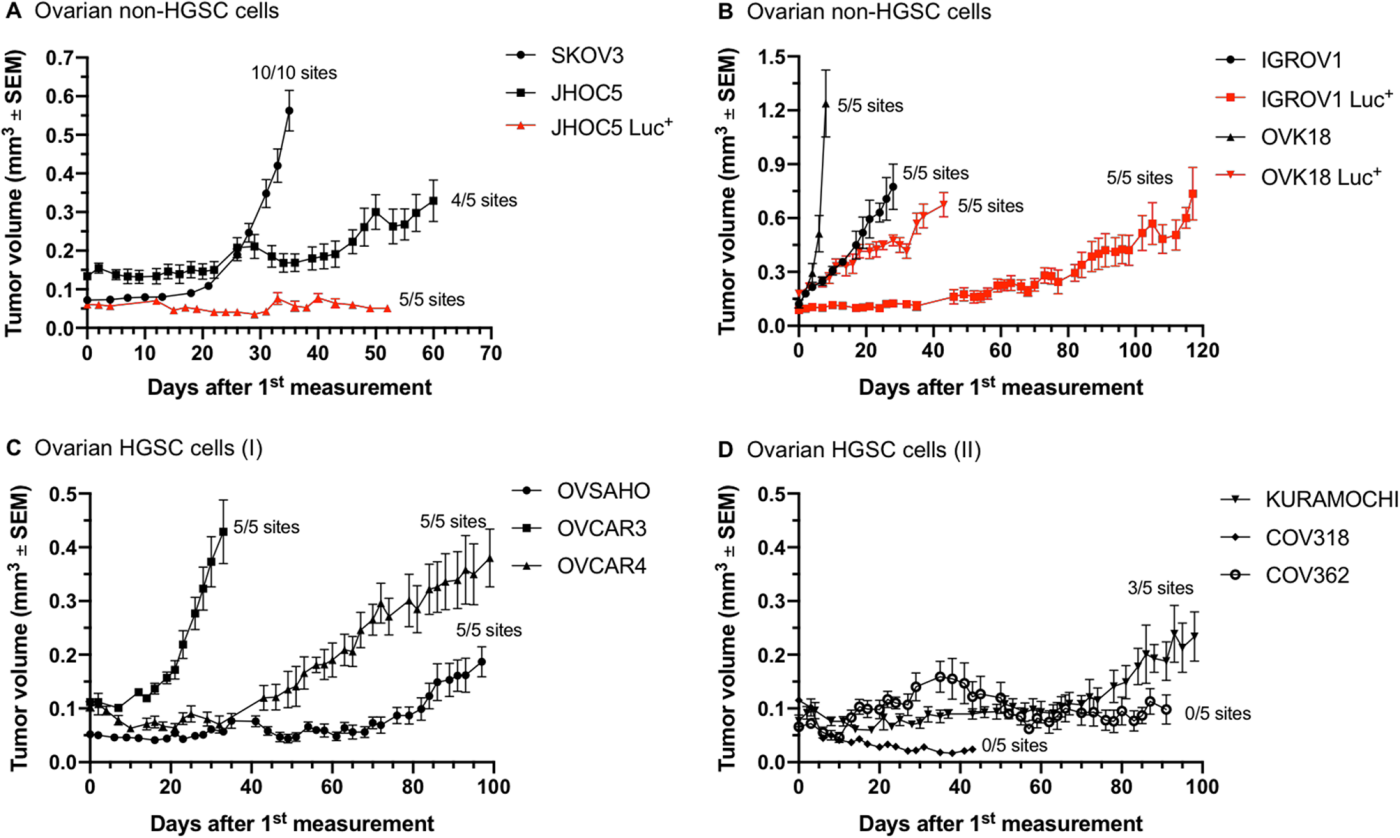
Growth of ovarian cancer cells as subcutaneous xenografts in female NSG mice. (**A**), (**B**) Ovarian non-HGSC cells. (**C**), (**D**) Ovarian HGSC cells. Curves in red depict growth of the Luc^+^ cell lines. For all experiments, five mice per group with unilaterally implanted tumors were used, except SKOV3 which was bilaterally implanted. *Luc*^*+*^ luciferase-expressing cells, *SEM* standard error of the mean.

While the ovarian non-HGSC cells, SKOV3, JHOC5, IGROV1, and OVK18 (Fig. 1A,B), produced xenografts robustly (JHOC5 tended to grow less efficiently in 4 out of 5 sites), we observed very different growth patterns for the HGSC cell lines (Fig. 1C,D). After an initial delay, OVCAR3 generated rapidly growing tumors, followed by OVCAR4 and OVSAHO that grew much more slowly. On the other end of the spectrum were KURAMOCHI cells, which demonstrated only a partial tumor take (3 out of 5 sites) and an extremely long lag phase before producing small tumors; and COV318 and COV362, the two cell lines that failed to grow as subcutaneous xenografts in female NSG mice.

In order to study ovarian tumor growth orthotopically, we also produced Luc-expressing versions of the ovarian cell lines, which we first tested for their growth in a subcutaneous location. All cell lines, with the exception of OVSAHO and OVCAR3, could be established to express Luc stably (Table 2). Injection of Luc-expressing versions of the cells (Luc^+^) alongside the parental cell lines revealed reduced tumor growth for the genetically altered cell lines of the non-HGSC type (compare JHOC5 vs. JHOC5 Luc^+^, Fig. 1A, and OVK18 vs. OVK18 Luc^+^, Fig. 1B). One exception was the IGROV1 Luc^+^ cell line that largely matched the in vivo growth kinetics of the parental IGROV1 cell line. Modification of the HGSC cell lines, OVCAR4 and KURAMOCHI, with the Luc abrogated their growth as subcutaneous xenografts (data not shown). Luc-transduced COV318 and COV362 did not produce any subcutaneous tumors similarly to the parental cell lines (data not shown).

Thus, unlike the non-HGSC xenograft models of ovarian cancer, only very few HGSC cell lines could be grown as subcutaneous tumors in the advanced immunocompromised NSG strain. They can be represented in the order of the efficiency with which they show tumorigenicity at a subcutaneous location: OVCAR3 > OVCAR4 > OVSAHO > KURAMOCHI > COV362 > COV318. With the exception of the OVCAR3 cell line, HGSC xenografts generally require a long engraftment time and result in smaller tumors. NSG mice tolerated the subcutaneous engraftment of ovarian cancer cells well, as judged by positive body weight dynamics (Supplementary Fig. S1).

### Growth of ovarian cancer cells as intraperitoneal xenografts in NSG mice

We next tested both parental and Luc^+^ ovarian cancer cell lines for their growth as xenografts in the peritoneal cavity of the female NSG mice, as a number of the HGSC cell lines have been successfully grown in this location, where they showed dissemination and histology reminiscent of the HGSC^22^. The results of this experiment are summarized in Table 3.

**Table 3:** Growth of ovarian cancer cells as intraperitoneal xenografts in female NSG mice.

Cell line	Type	Tumorigenic?	Days to license limit	Ascites?
**Parental cell lines**				
JHOC5	Non-HGSC	Yes (5/5 mice)	~ 25	Yes
IGROV1	Non-HGSC	Yes (5/5 mice)	~ 10	No
OVK18	Non-HGSC	Yes (5/5 mice)	~ 21	Yes
COV318	HGSC	No (0/5 mice)	n.g.	n.g.
COV362	HGSC	Partially (3/5 mice)	~ 100	Yes
KURAMOCHI	HGSC	No (0/5 mice)	n.g.	n.g.
OVCAR4	HGSC	Yes (4/5 mice)	~ 150	No
OVSAHO	HGSC	Partially (5/10 mice)	~ 120	Yes
**Cells expressing luciferase (Luc**^+^**)**				
JHOC5 Luc^+^	Non-HGSC	Yes (5/5 mice)	~ 26	Yes
SKOV3 Luc^+^	Non-HGSC	Yes (5/5 mice)	~ 38	Yes
IGROV1 Luc^+^	Non-HGSC	Yes (5/5 mice)	~ 33	No
OVK18 Luc^+^	Non-HGSC	Yes (5/5 mice)	~ 28	Yes
COV318 Luc^+^	HGSC	Yes (5/5 mice) – ascites model	~ 43	Yes
COV362 Luc^+^	HGSC	Yes (5/5 mice)	~ 89	Yes
KURAMOCHI Luc^+^	HGSC	No (0/5 mice)	n.g.	n.g.
OVCAR4 Luc^+^	HGSC	Partially (2/5 mice)	~ 150	No

Cell line types presented are after Domcke et al.^21^.

*HGSC* high-grade serous carcinoma, *Luc*^*+*^ luciferase-expressing cells, *n.g.* no growth.

**Figure 2:**
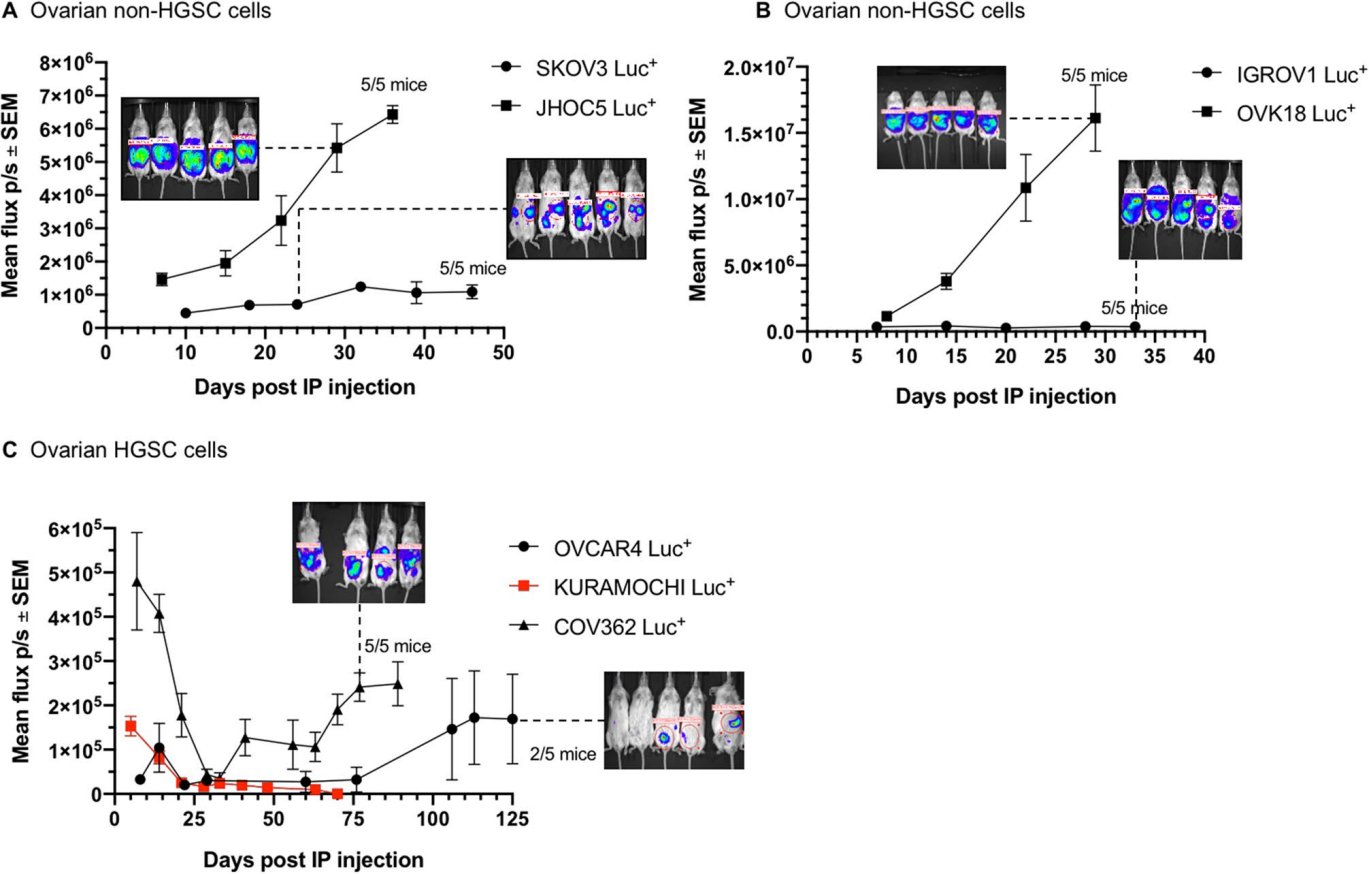
Growth of ovarian cancer cells as intraperitoneal xenografts in female NSG mice. (**A**), (**B**) Ovarian non-HGSC cells. (**C**) Ovarian HGSC cells. Note to **C**: The inset containing the IVIS image (taken on Day 75 post IP injection) shows surviving 4 of originaly 5 mice carrying intraperitoneal COV362 Luc^+^ tumors. Curve in red indicates the regression of KURAMOCHI tumors. For all experiments, five mice per group were used. *IP* intraperitoneal, *Luc*^*+*^ luciferase-expressing cells, *p/s* per second, *SEM* standard error of the mean.

Of the non-HGSC cell lines, SKOV3 and JHOC5, formed intraperitoneal tumors with ascites. The intraperitoneal injection of SKOV3 Luc^+^ cells resulted in small tumors surrounding the pancreas/omentum/mesenteries (Supplementary Fig. S2A), while JHOC-5 (parental and Luc^+^ cell lines) produced small tumors throughout the abdomen (Supplementary Fig. S2B). Following the tumor bioluminescence by IVIS revealed rapid development of JHOC5 tumors, while SKOV3 tumor growth kinetics did not greatly change once intraperitoneal tumors had been established (Fig. 2A). OVK18 (both parental and Luc^+^ cell lines) formed rapidly growing intraperitoneal tumors with ascites (Supplementary Fig. S2C). Parental IGROV1 grew more slowly as intraperitoneal xenografts (Supplementary Fig. S2D) than IGROV1 Luc^+^ cells (Fig. 2B; Table 3). Also, in contrast to OVK18 Luc^+^, neither of IGROV1 cell lines produced ascites, and the low bioluminescent signal of IGROV1 Luc^+^ did not change substantially during the course of the experiment (Fig. 2B).

Only few HGSC cell lines were tumorigenic in the peritoneal cavity of the female NSG mice (Table 3; OVCAR3 was not tested as an intraperitoneal xenograft model). Thus, of the parental (untransduced) cell lines, the intraperitoneal injection of COV362, OVCAR4, and OVSAHO resulted in small tumors spread across the peritoneal cavity (Supplementary Fig. S2E), while COV362 and OVSAHO also produced ascites. Parental COV318 and KURAMOCHI cell lines were not tumorigenic under the chosen conditions. Interestingly, there were some differences observed with the Luc-transduced versions of the HGSC cells, with COV318 Luc^+^ cells gaining tumorigenicity by growing as a fine film on internal organs (Supplementary Fig. S2F) and producing ascites-borne tumor cell suspension (that could be visualized by centrifugation of the drained ascitic fluid) that however failed to produce a consolidated bioluminescent signal when imaged by IVIS (data not shown). Also, COV362 Luc^+^ cells showed somewhat improved tumor take in the peritoneum, which could be revealed after a long lag time (Fig. 2C). On the other hand, OVCAR4 Luc^+^ cells showed reduced tumorigenicity, with only 2 out of 5 mice showing the bioluminescent signal (Fig. 2C). After initial dwelling in the peritoneum, KURAMOCHI Luc^+^ cells were cleared from the NSG mice by Day 70 post injection (Fig. 2C).

Thus, the orthotopic injection of the ovarian cells into the peritoneal cavity of the NSG mice may have only marginally improved the tumorigenic properties of some HGSC cell lines (such as COV318 Luc^+^, which grew as a film and cell suspension in the ascitic fluid, and COV362 Luc^+^, which grew as small solid tumors), while revealing the potent ascites-forming capacity of many cell lines across the spectrum of ovarian cancer models (Table 3). The intraperitoneal models of ovarian cancer were generally well tolerated by female NSG mice, as judged by the steady gain in body weight (Supplementary Fig. S3). However, the build-up of the abdominal ascitic fluid also contributed to the body weight gains, as illustrated by the marked change in body weight after ascites drainage (Supplementary Fig. S3D). This needs to be considered when setting up intraperitoneal xenograft models of ovarian cancer.

### Growth of HGSC cells as intrabursal xenografts in NSG mice

Given the limited success of the intraperitoneal models, we also tested several of the ‘more difficult’ HGSC cell lines as orthotopic models using the ovarian bursa of the mice, which may arguably represent a better environment for the autochthonous growth of some ovarian cancers. Small numbers (5 × 10^4^ cells) of parental and Luc^+^ cells, COV362 and KURAMOCHI, were injected into the surgically exposed bursa of the female NSG mice, and the animals were monitored for general well-being, change in body weight, and ascites. Luc^+^ cell lines were also monitored by IVIS. The results of this study are summarized in Table 4.

**Table 4:** Growth of ovarian cancer cells as intrabursal xenografts in female NSG mice.

Cell line	Type	Tumorigenic?	Days to license limit	Ascites?
**Parental cell lines**				
COV362	HGSC	Partially (2/5 mice)	~ 180	No
KURAMOCHI	HGSC	No (1/5 mice)	~ 200	No
**Cells expressing luciferase**				
COV362 Luc^+^	HGSC	Partially (2/3 mice)	~ 180	No
KURAMOCHI Luc^+^	HGSC	No (0/5 mice)	n.g.	No

Cell line types presented are after Domcke et al.^21^.

*HGSC* high-grade serous carcinoma, *Luc*^*+*^ luciferase-expressing cells, *n.g.* no growth.

**Figure 3:**
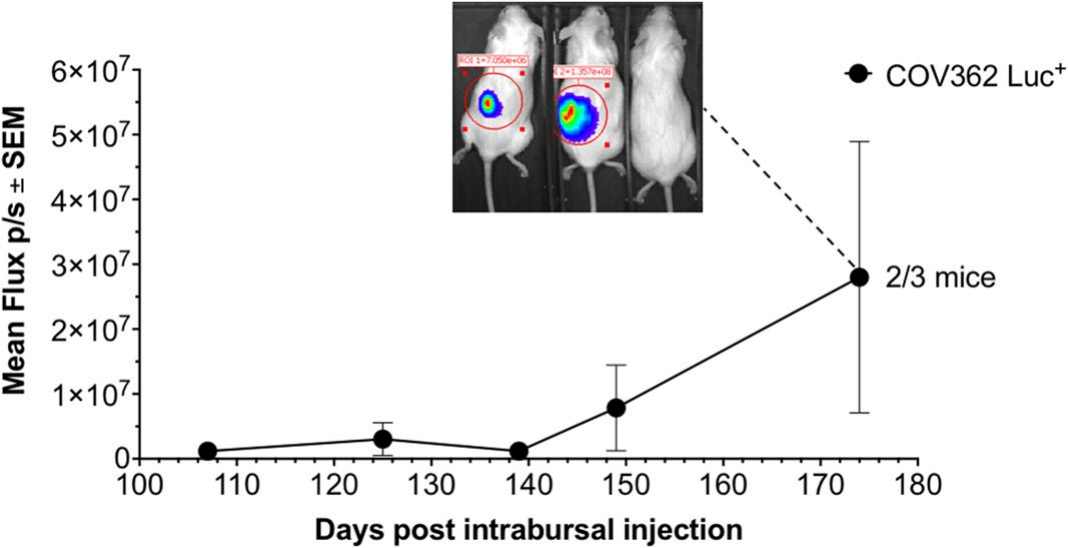
Growth of HGSC COV362 Luc^+^ cells as intrabursal xenografts in female NSG mice. For all experiments, three mice per group were used. *Luc*^*+*^ luciferase-expressing cells, *p/s* per second, *SEM* standard error of the mean.

One of the animals with the intrabursal injection of parental KURAMOCHI cells developed a tumor (Supplementary Fig. S4C), while KURAMOCHI Luc^+^ line failed to develop tumors (data not shown). On the other hand, both COV362 and COV362 Luc^+^ tumors could be observed approximately 100 days post injection in some animals (Fig. 3; Supplementary Fig. S4A and Fig. S4B).

These results suggest that the autochthonous location alone is not likely to improve the tumor growth characteristics of the HGSC cell lines in the highly immunocompromised mice in vivo. Body weight curves demonstrate that NSG mice, in which the establishment of the intrabursal model was successful, tolerate intrabursal xenografts well (Supplementary Fig. S5).

### Profiling ovarian cancer cells for ERα expression

**Figure 4:**
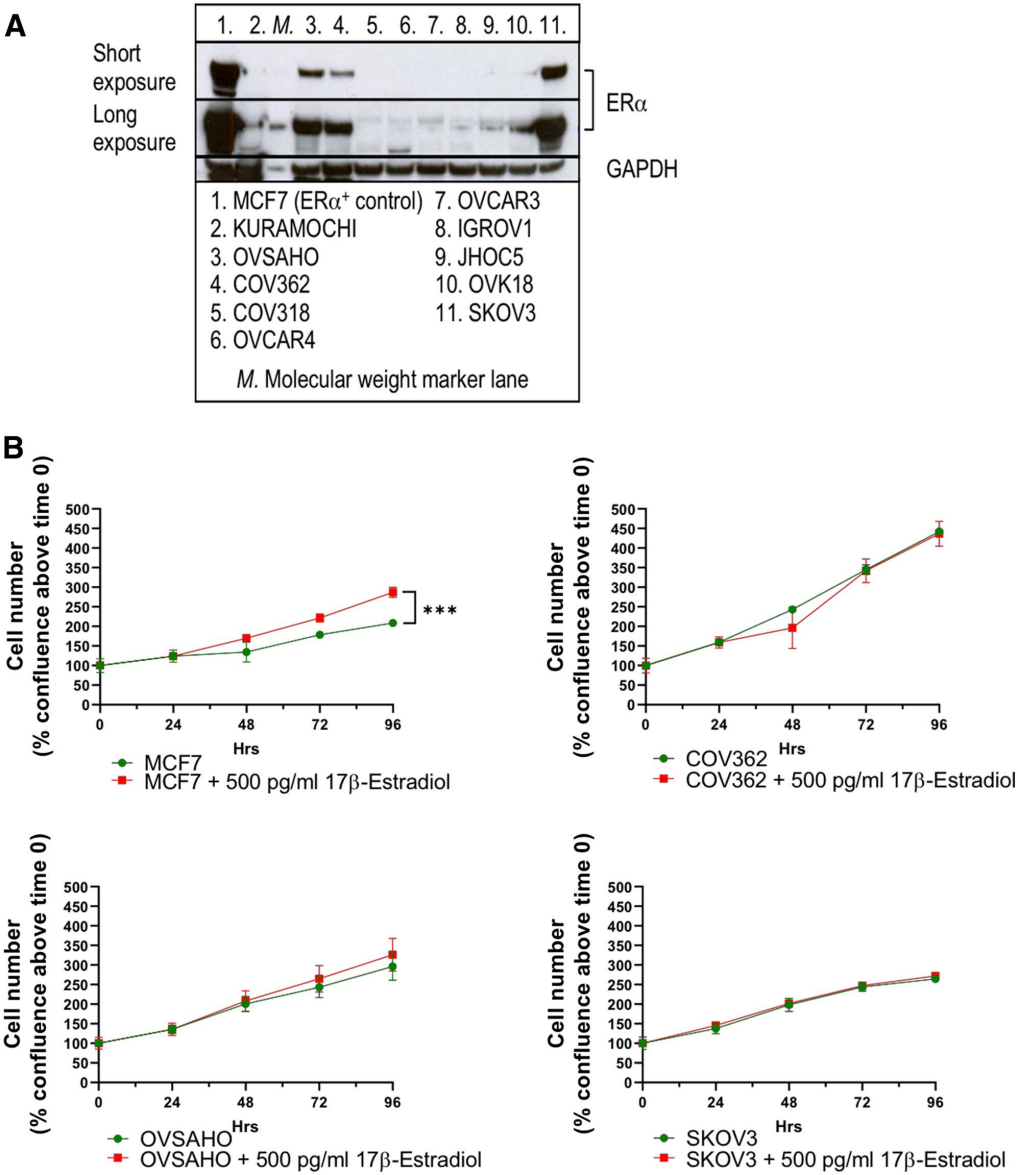

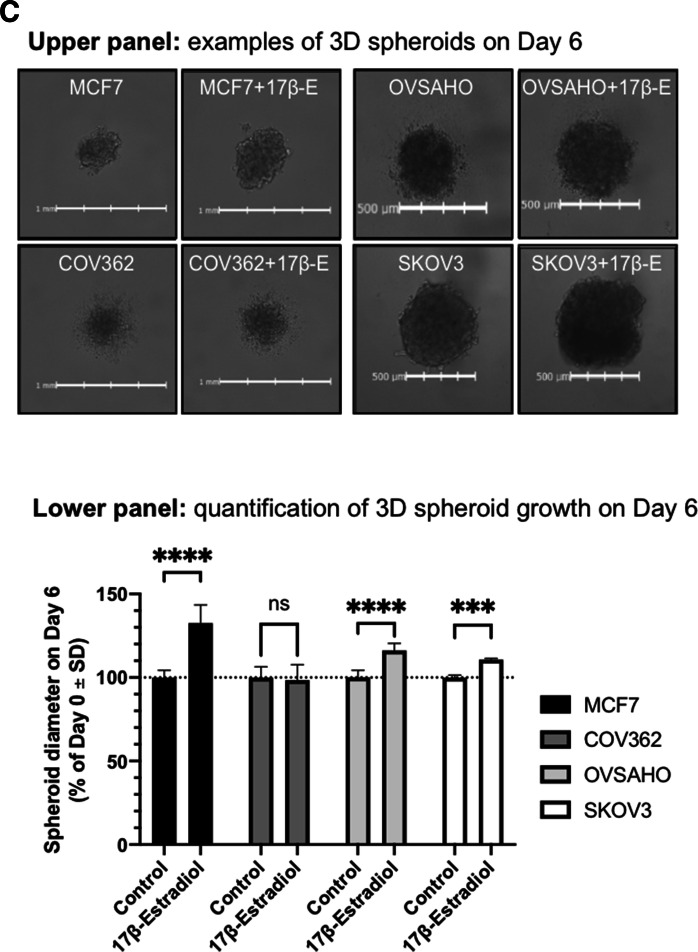
Characterization of ovarian cancer cell lines for their estrogen sensitivity in vitro. (**A**) Profiling of ovarian cancer cells by Western blot. The panels shown were obtained from a larger set of Western blot, digitally cropped to display the relevant bands (see Fig. S7 for the complete set of blots used in the experiment). (**B**) Response of ER⍺^+^ cells to 17β-Estradiol (2D assay); cell counts were performed in triplicates. (**C**) Response of ER⍺^+^ cells to 17β-Estradiol (3D assay); individual spheroids from 6 wells per cell line per condition were analyzed. Upper panel: examples of 3D spheroids on Day 6. Lower panel: quantification of 3D spheroid growth on Day 6 (MCF7 with 17β-Estradiol: diameter range 428–492 µm, without 17β-Estradiol: 331-366 µm, p ≤ 0.0001; COV362 + 17β-Estradiol: diameter range 507–568 µm, without 17β-Estradiol: 496–581 µm, not significant; OVSAHO + 17β-Estradiol: diameter range 480–515 µm, without 17β-Estradiol: 407–451 µm, p ≤ 0.0001; SKOV3 + 17β-Estradiol: diameter range 617–635 µm, without 17β-Estradiol: 558–573 µm, p = 0.0003). Statistical analysis was performed using unpaired two-tailed *t* test (GraphPad Prism 8). *ERα* estrogen receptor α, *ns* not significant, *SD* standard deviation.

Human ovarian cancer cells have been described to express hormone receptors^24,25^. We tested the expression of ERα in the select ovarian cancer cells (Table 1) by Western blot, while using the well-known ERα^+^ breast cancer cell line MCF7 as a positive control. The expression of ERα strongly varied amongst the different cell lines, with the highest levels (after the positive control MCF7 cells) detected in SKOV3 and the HGSC cells, OVSAHO and COV362 (Fig. 4A).

### Testing sensitivity of ovarian cancer cells to the addition of 17β-Estradiol in vitro

The finding that several ovarian cancer cell lines, including those of the HGSC origin, were strongly positive for ERα prompted us to test whether their growth in vitro can be influenced by the addition of 17β-Estradiol into the medium. We used 500 pg/ml 17β-Estradiol^30^, the free plasma concentration that is usually observed in animals that receive subcutaneous implants consisting of 0.36-mg 17β-Estradiol pellets (see “Materials and methods”). While the 2D growth of the control cell line, MCF7, was clearly stimulated by the addition of the hormone, no statistically significant changes in the proliferation rates were observed for the ERα^+^ ovarian cancer cells (Fig. 4B).

It is known that cancer cells show great differences in their transcriptional activity, signal transduction, and metabolism, as well as the response to drugs when grown under 3D rather than 2D conditions^31^. We established growth conditions for 3D ovarian cancer cell spheroids using ULA plates (Fig. 4C). Both OVSAHO and SKOV3 formed relatively tight spheroids on Day 6 post seeding, which became slightly (but statistically significantly) larger when grown in the presence of 17β-Estradiol. MCF7 cells formed spheroids inefficiently; they, however, showed some response to 17β-Estradiol by increasing the size of the spheroid-like structures. COV362 cells failed to establish spheroids, and also the addition of 17β-Estradiol could not stimulate spheroid formation under the ULA plate conditions for this HGSC cell line. The 3D-spheroid assay data however revealed that the ERα expression correlates with the increased proliferation of MCF7, OVSAHO, and SKOV3 cells in the presence of the ERα ligand, 17β-Estradiol.

### Testing sensitivity of ovarian cancer xenografts to the addition of 17β-Estradiol in vivo

The modest response of the ERα^+^ breast and ovarian cancer cell lines to estrogen supplementation in vitro (especially under 3D conditions) prompted us to test whether estrogen can help establish HGSC xenografts in the highly immunocompromised NSG mice. We injected COV362 and OVSAHO cells subcutaneously into the flank of ovariectomized mice that had also been implanted with a 60-day 17β-Estradiol pellet. MCF7 cells were used as a well-described positive control of an estrogen-dependent xenograft model, while OVCAR4 were used as a negative control (these cells showed an unusual low-molecular weight band for ERα-specific antibodies in Western blot, Fig. 4A). The non-HGSC cell line, SKOV3, was found to express large amounts of ERα (Fig. 4A) and was also tested for responsiveness to the addition of the hormone.

**Figure 5:**
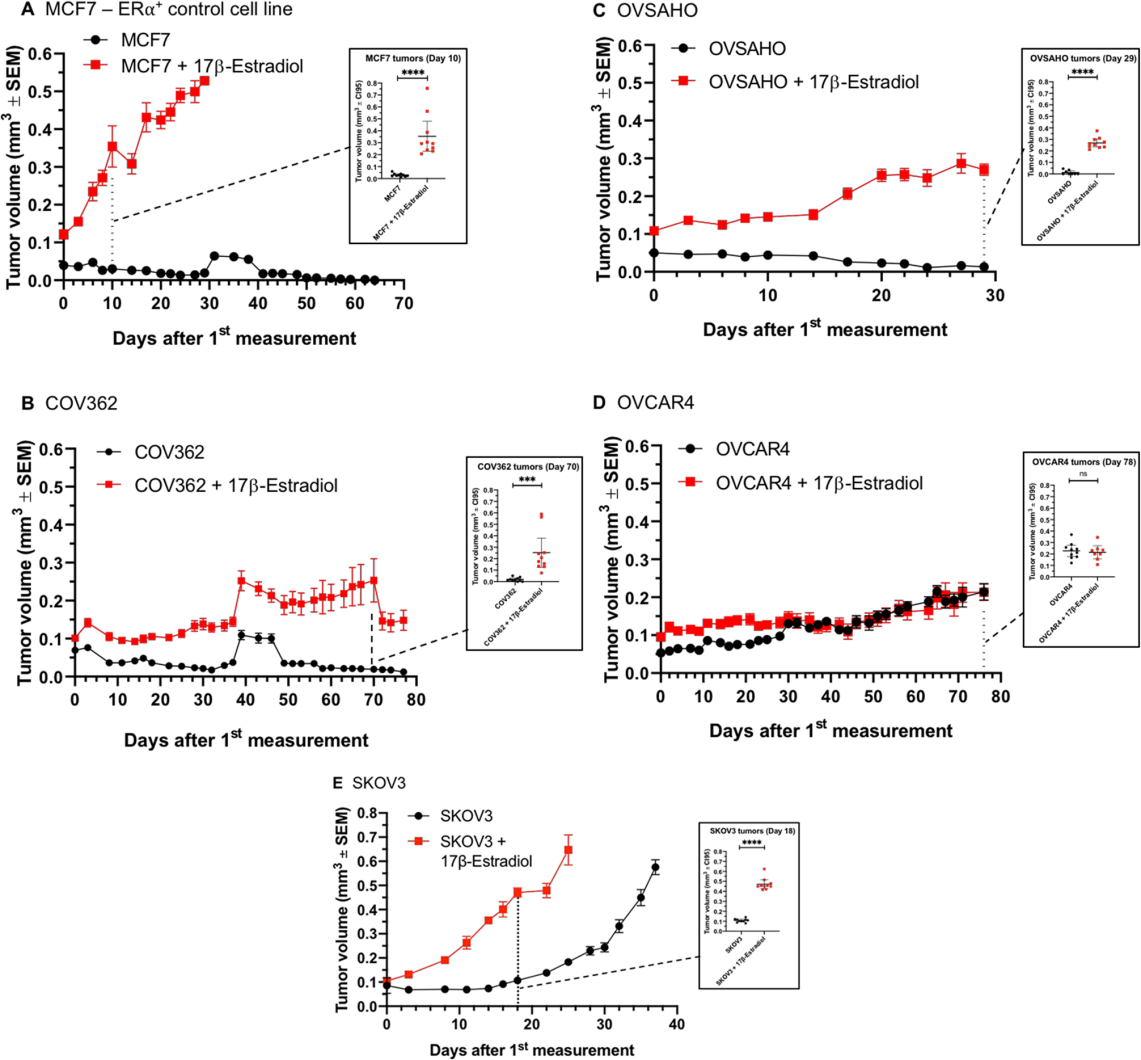
Characterization of ovarian cancer cell lines for their estrogen sensitivity in vivo. (**A**) MCF7, ER⍺^+^ control cell line (Day 10, p ≤ 0.0001). (**B**) COV362 (Day 70, p = 0.0006). (**C**) OVSAHO (Day 29, p ≤ 0.0001). (**D**) OVCAR4 (Day 76, not significant). (**E**) SKOV3 (Day 18, p ≤ 0.0001). For all experiments, five mice with unilaterally implanted tumors were used. Statistical analysis was performed using unpaired two-tailed *t* test (GraphPad Prism 8). *SEM* standard error of the mean.

As shown on Fig. 5A, the control cell line MCF7 showed a clear dependence on the presence of estrogen in the ovariectomized NSG mice, and no MCF7 tumors could be observed in the control animals without the hormone pellet. Strikingly, the tumor take for COV362 could be dramatically improved by the estrogen supplementation (compare Figs. 1D and 5B), even though the model remains a rather slow one. Interestingly, even after 60 days (after which the release of 17β-Estradiol from the pellet ceases), the subcutaneous COV362 tumors kept steady growth, suggesting the gain of proliferative capacity in the tumors once they have become established. The OVSAHO model was the second clearly estrogen-sensitive xenograft model in vivo, with tumors growing rapidly in the presence of the constant supply of 17β-Estradiol (Fig. 5C). In contrast, the ER^-^ HGSC cell line, OVCAR4, did not show any dependence on estrogen for its growth and was growing steadily and slowly in the subcutaneous location (Fig. 5D; also compare with Fig. 1C).

Of interest, the well-characterized non-HGSC SKOV3 xenograft model showed remarkable sensitivity to estrogen for its growth as a subcutaneous xenograft in the ovariectomized mice (Fig. 5E). The SKOV3 tumor growth pattern strongly correlated with the ERα^+^ status of this cell line and, together with the OVSAHO and COV362 data, suggests a much broader role for hormone signaling across the different types of ovarian cancer. Supplementation with 17β-Estradiol at the chosen dose did not have a negative effect on the mouse wellbeing as judged by no major body weight loss observed in the study (Supplementary Fig. S6).

## Discussion

In this study, we systematically assessed the growth of a panel of human ovarian cancer cell lines as subcutaneous and intraperitoneal tumor xenografts. In contrast to the published work that mostly used nude mice^22,32,33^ (while Elias et al. only assessed the growth of two HGSC cell lines in NSG mice^23^), we investigated the growth of ovarian cancer xenografts in the highly immunodeficient NSG mice. We confirmed that the unmodified (parental) non-HGSC cell lines, SKOV3, JHOC5, IGROV-1 and OVK-18, readily form tumors when injected either subcutaneously or intraperitoneally (Fig. 1A,B). Viral transduction of JHOC5, IGROV1, and OVK18 cells with the firefly luciferase negatively impacted the ability of the cell lines to grow as subcutaneous tumors (Fig. 1A,B). It is presently not clear why the luciferase transduction would interfere with the growth of ovarian xenografts in the subcutaneous location. Previous studies using luciferase-positive ovarian cells did not directly compare the growth of parental and engineered cells^23^. While formally this could represent a clonal effect in vivo, lentiviral transduction efficiency was so high with the majority of the cell lines that clonal selection of Luc^+^ cells in vitro was negligible. Prior to injection into mice, Luc^+^ cells were verified for their identity using STR profile so that we can exclude artefacts linked to potential cell mislabeling. Availability of luciferase-positive cells enabled following the progress of the intraperitoneal tumors, which could be produced by the non-HGSC cell lines. Ascites was a frequent feature associated with the intraperitoneal ovarian tumors as also observed previously^32^.

In stark contrast, of the six validated HGSC cell lines^21^ injected subcutaneously, only two unmodified cell lines produced satisfactory tumor growth in this location. OVCAR3 is one clear exception that gave sizable tumors within 40 days of injection, while OVCAR4 is a slow growing tumor model (Fig. 1C). These results fit very well earlier observations made by Mitra et al. in nude mice^22^. Of interest, other HGSC cell lines found at the top of the list published by Domcke et al.^21^, i.e. KURAMOCHI, OVSAHO, COV318, and COV362, invariably failed to grow robustly as subcutaneous xenografts in the NSG mice (Fig. 1C,D). This seems to be at odds with the study of Elias et al. who reported some success with KURAMOCHI and OVSAHO xenografts in nude mice (albeit the tumors in their studies showed variable and somewhat unreliable growth rates). Since they used luciferase-modified cells in their in vivo study, some effect of clonal selection on growth properties of the HGSC cells cannot be excluded^23^.

Injection of Luc^+^ HGSC cells into the peritoneum of the mice did not significantly improve the tumor take rate for the HGSC cells, while COV362 Luc^+^ cells did form solid tumors after a long period of regression and latency (Fig. 2C). This model produced significant amount of ascites, which is at odds with the previous study by Mitra et al. who did not observe fluid buildup in the intraperitoneal COV362 model in the nude mice^22^. A very interesting ascites HGSC model in our hands is represented by COV318 Luc^+^ cells that form a film rather than a solid tumor (Supplementary Fig. S2F) and could be plentifully retrieved from the ascitic fluid collected from the peritoneal cavity of the NSG mice previously injected with the tumor cells (data not shown).

We tested the growth of the parental and Luc^+^ versions of KURAMOCHI and COV362 as autochthonous tumor models by injecting them into the ovarian bursa of female NSG mice. While some mice allowed the establishment of COV362 tumors (Fig. 3; Supplementary Fig. S4A and S4B), KURAMOCHI remained largely refractory to tumor growth under these conditions, with only one mouse developing a sizable tumor (Supplementary Fig. S4C). Given the slow growth of intrabursal models, in part due to the low number of cells that can be injected, and the technically demanding procedure, and also, given the lack of significant improvement in the growth rates of the COV362 model, we doubt this type of a HGSC model will gain popularity as a robust model especially in preclinical drug development.

In the pursuit to establish more robust preclinical models for HGSC and to complement the OVCAR3 and OVCAR4 models that may well be suitable for the future HGSC modeling work (Fig. 1C), we investigated whether estrogen supplementation could enhance the growth of some of the ovarian cancer cells in vivo. We could clearly differentiate the given panel of ovarian cancer cells by ERα expression, with two HGSC cell lines, OVSAHO and COV362, and one non-HGSC cell line, SKOV3, demonstrating relatively high levels of the hormone receptor expression (Fig. 4A). In vitro data revealed that 17β-Estradiol supplementation at levels relevant for in vivo studies could, to some extent, stimulate the formation of 3D spheroids by the ERα^+^ cells (Fig. 4C). Importantly, 17β-Estradiol supplementation in the mice allowed the establishment of both OVSAHO and COV362 xenografts in a subcutaneous location (Fig. 5A,B), thus expanding the panel of usable HGSC models to four: OVCAR3, OVCAR4, OVSAHO, and COV362.

Interestingly, the well-studied SKOV3 model proved to be estrogen-sensitive in our hands (Fig. 5E). Previously, a 32-bp deletion in exon 1 in ERα was identified in this cell line^34^ and used to explain why SKOV3 had not been found estrogen-responsive in vitro in an earlier study^35^. We did not observe estrogen sensitivity of SKOV3 in 2D culture (Fig. 4B). However, this was different when the cells were grown as 3D spheroids (Fig. 4C) and subcutaneous tumors (Fig. 5E). Our finding thus suggests much broader involvement of the hormone signaling across different types of ovarian cancer, which deserves dedicated study using advanced models. For instance, a recent study described a paradoxical effect of the 17β-Estradiol supplementation that improved subcutaneous growth of OVCAR3 tumors, which generally lacks expression of ERα^36^. Other known estrogen-sensitive ovarian cancer cells include PEO4, which further expands the repertoire of estrogen-sensitive ovarian cancer models^37^.

Considerably more work is required to assess the utility of 17β-Estradiol supplementation for HGSC and other forms of ovarian cancer, so that broader use of hormone supplementation may help expand the repertoire of difficult-to-grow ovarian cancer models. One limitation of 17β-Estradiol pellets is that, at high 17β-Estradiol levels, they may lead to renal damage and urosepsis in mice^30,38^. Thus, significant care must be exercised when escalating the dose of 17β-Estradiol for ovarian tumor modelling work. At the doses tested, we did not observe any detrimental effect of 17β-Estradiol pellets on ovariectomized female NSG mice (Supplementary Fig. S6).

Another potential caveat of hormone supplementation in preclinical models in vivo will undoubtedly be its physiological and supraphysiological effects on tumor cells. Ligand-bound ERα recognizes over 70,000 estrogen-response elements (EREs) in the human and mouse genomes^39^ and will directly affect transcription of a host of genes. Estrogen receptors also modulate gene transcription via an indirect mechanism by stabilizing DNA–protein complexes and/or recruiting co-activators, impinging on cell signaling mediated by e.g. AP-1 and NFκB transcription factors (reviewed in^40^). This pleiotropic effect of estrogen will therefore potentially cause an altered response to a therapy on the part of ovarian tumors grown in the animal model. While a number of ER^+^ breast cancer models, such as MCF-7 that require in vivo estrogen supplementation (reviewed in^41^), are routinely used in drug development, future work will need to investigate the effect of estrogen supplementation on ovarian cancer cell biology and response to targeted therapeutics.

## Supplementary information

Supplementary Information 1

Supplementary Information 2

## Abbreviations

HGSC: High-grade serous carcinoma
IP: Intraperitoneal
IVIS: In vivo imaging system
LGSC: Low-grade serous carcinoma
Luc: Luciferase
NOD: Non-obese diabetic
NSG: NOD scid gamma
PD: Pharmacodynamics
PK: Pharmacokinetics
SC: Subcutaneous
scid: Severe combined immunodeficiency
STR: Short tandem repeat
