# Supplementary material for: Identification of ovarian high-grade serous carcinoma cell lines that show estrogen-sensitive growth as xenografts in immunocompromised mice
Source: Sci Rep. 2020 Jul 1;10:10799. doi: 10.1038/s41598-020-67533-1 (PMC7329846; doi:10.1038/s41598-020-67533-1)
Supplement: Supplementary file 1 — Supplementary Information 1 [file 41598_2020_67533_MOESM1_ESM.pdf]

Identification of ovarian high-grade serous carcinoma cell lines that show estrogen-sensitive growth as xenografts in immunocompromised mice

Alexis De Haven Brandon[1], Gary Box[1], Albert Hallsworth[1], William Court[1], Nicoll Matthews[1], Balint Herodek[1], Aitor Bermejo Arteagabeitia[1], Melanie Valenti[1], Vladimir Kirkin[1,*]

[1]Cancer Research UK Cancer Therapeutics Unit, The Institute of Cancer Research,

London SM2 5NG, UK

*Correspondence to vladimir.kirkin@icr.ac.uk

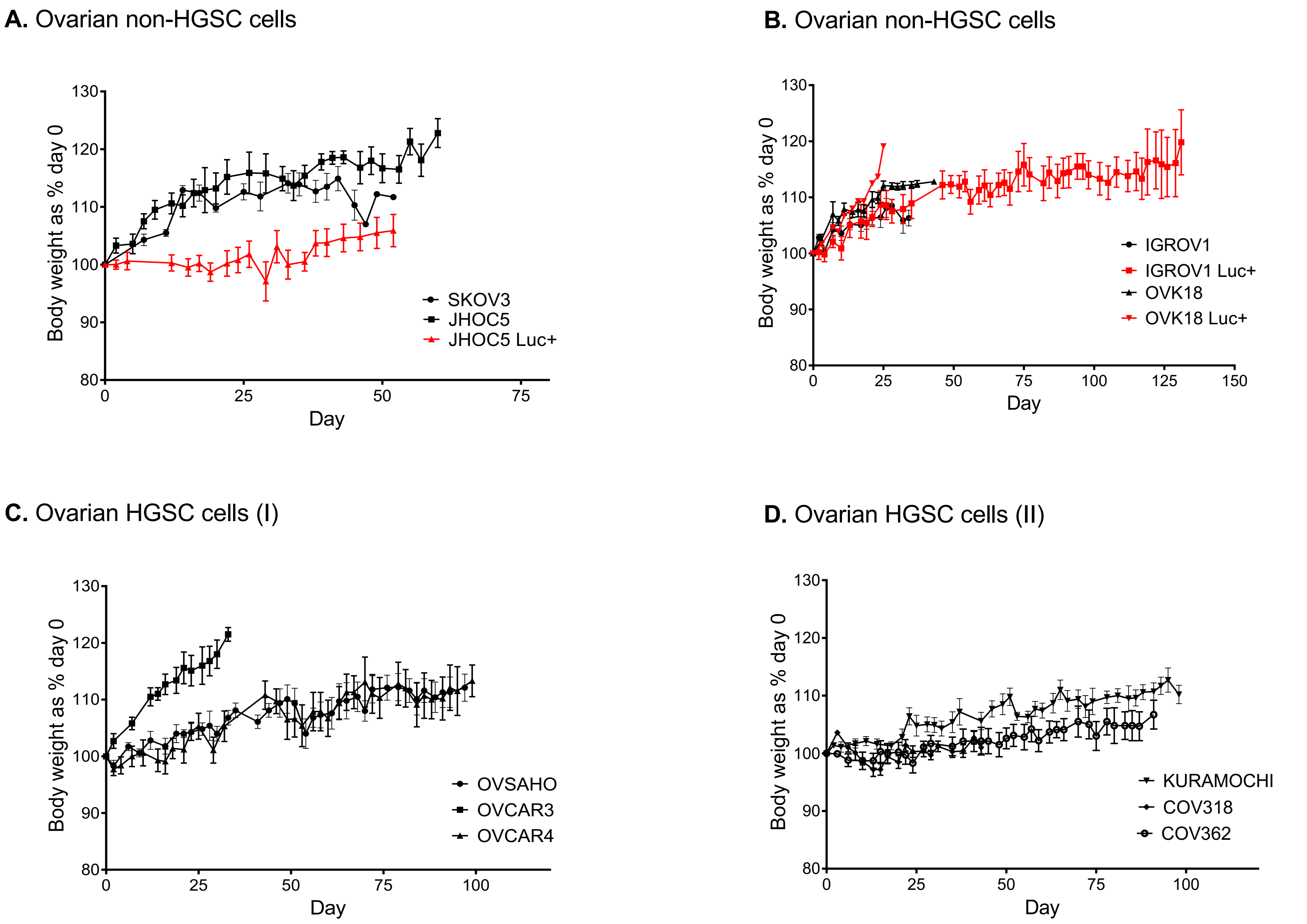
A. Ovarian non-HGSC cells
Body weight as % day 0
130
120
110
100
90
80
0
25
50
75
Day
SKOV3
JHOC5
JHOC5 Luc+
B. Ovarian non-HGSC cells
Body weight as % day 0
0
25
50
75
100
125
150
Day
IGROV1
IGROV1 Luc+
OVK18
OVK18 Luc+
C. Ovarian HGSC cells (I)
Body weight as % day 0
Day
OVSAHO
OVCAR3
OVCAR4
D. Ovarian HGSC cells (II)
Body weight as % day 0
Day
KURAMOCHI
COV318
COV362

Figure S1

A. Ovarian non-HGSC cells – SKOV3 Luc^{+} cells

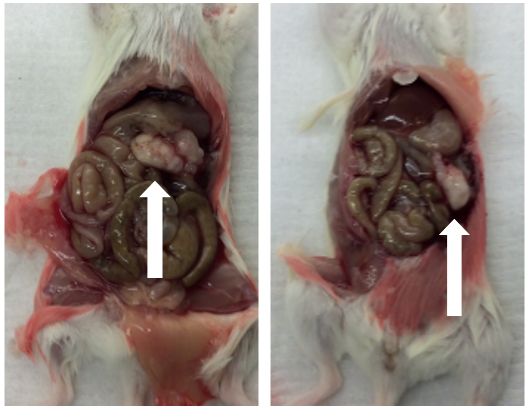

B. Ovarian non-HGSC cells – JHOC5 cells

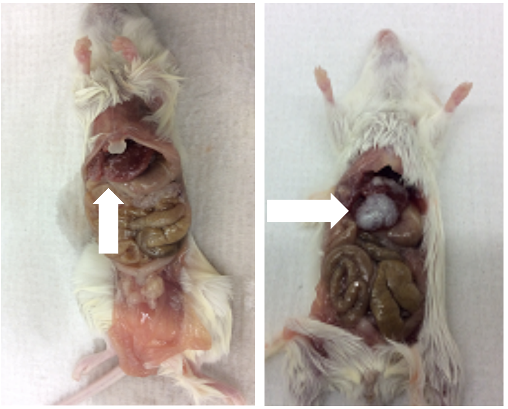

C. Ovarian non-HGSC cells – OVK18 cells

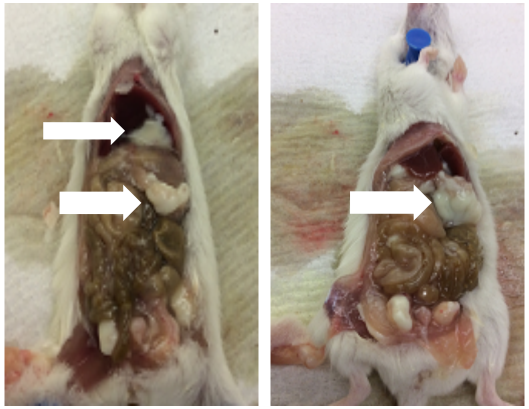

D. Ovarian non-HGSC cells – IGROV1 cells

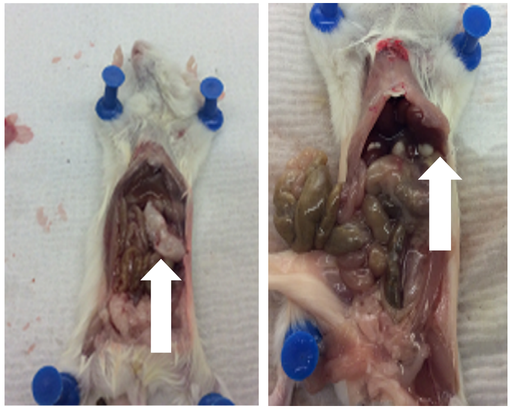

E. Ovarian HGSC cells – OVCAR4 cells

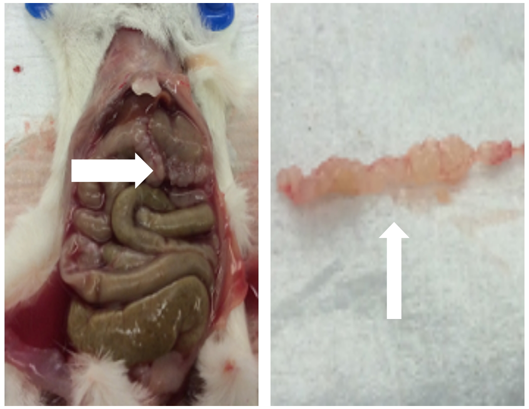

F. Ovarian HGSC cells – COV318 Luc^{+} cells

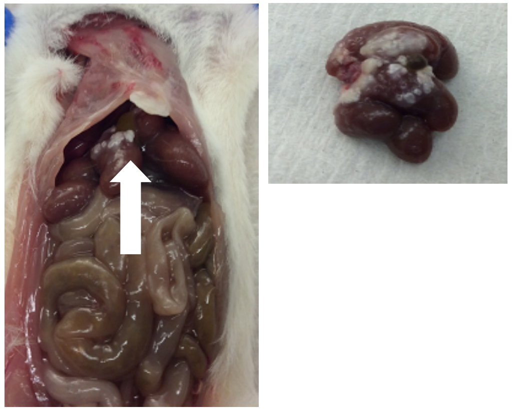

Figure S2

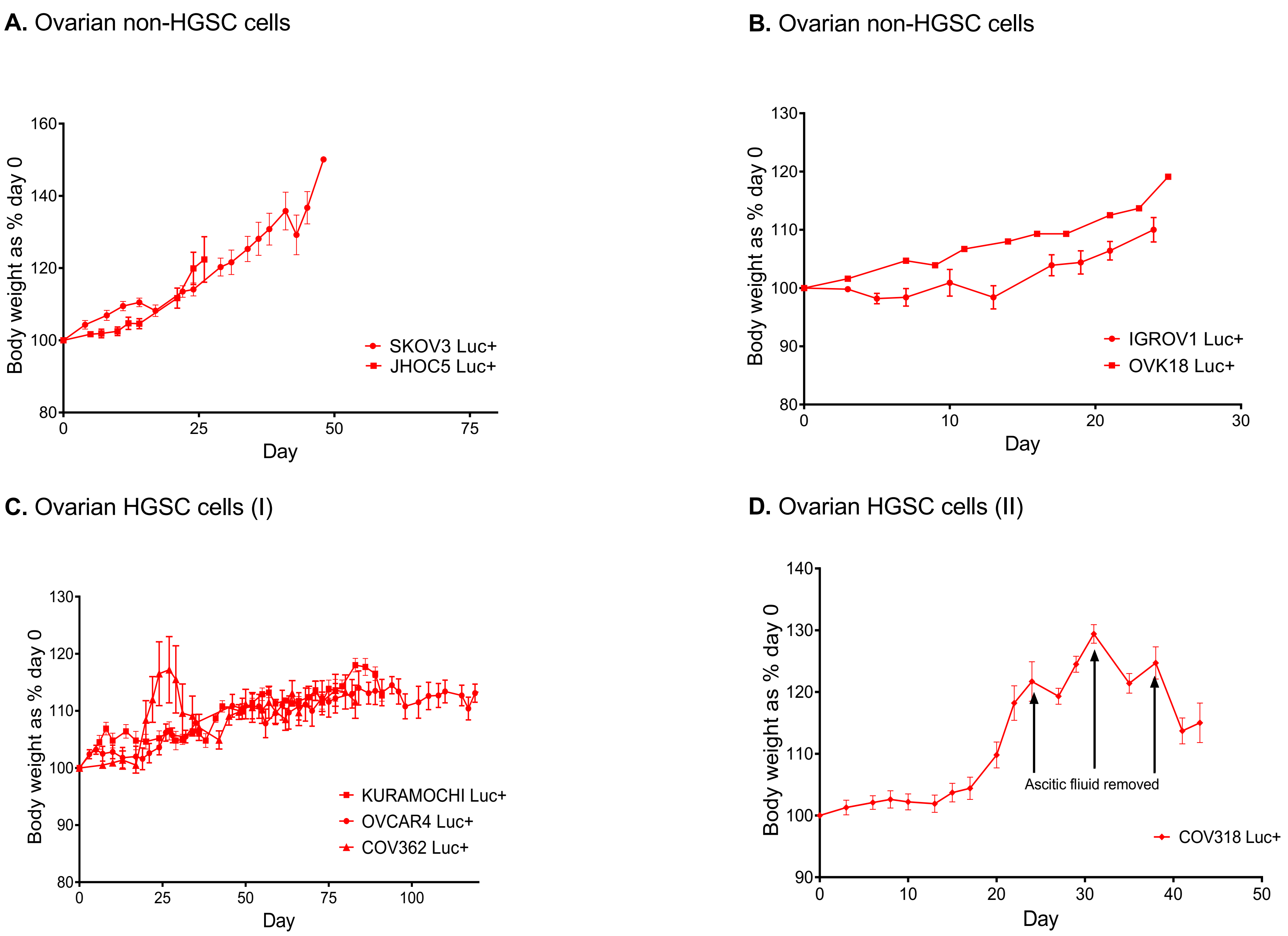
A. Ovarian non-HGSC cells
Body weight as % day 0
160
140
120
100
80
0
25
50
75
Day
SKOV3 Luc+
JHOC5 Luc+
B. Ovarian non-HGSC cells
Body weight as % day 0
130
120
110
100
90
80
0
10
20
30
Day
IGROV1 Luc+
OVK18 Luc+
C. Ovarian HGSC cells (I)
Body weight as % day 0
130
120
110
100
90
80
0
25
50
75
100
Day
KURAMOCHI Luc+
OVCAR4 Luc+
COV362 Luc+
D. Ovarian HGSC cells (II)
Body weight as % day 0
140
130
120
110
100
90
0
10
20
30
40
50
Day
Ascitic fliuid removed
COV318 Luc+

Figure S3

A. Ovarian HGSC cells – COV362 cells

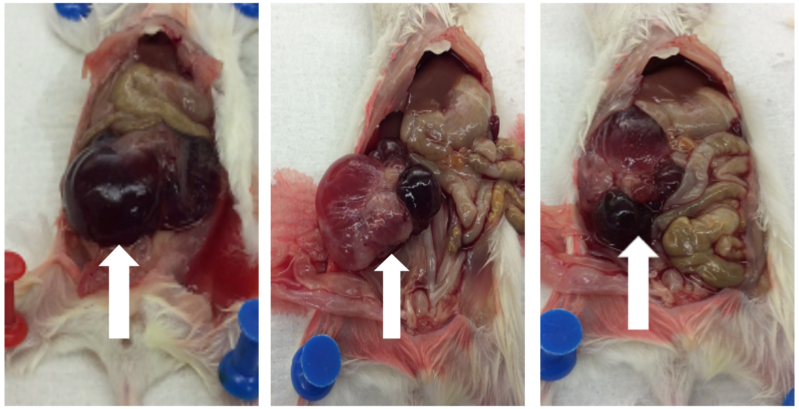

B. Ovarian HGSC cells – COV362 Luc^{+} cells

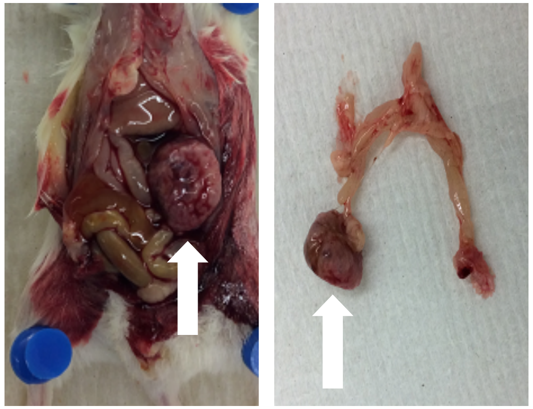

C. Ovarian HGSC cells – KURAMOCHI cells

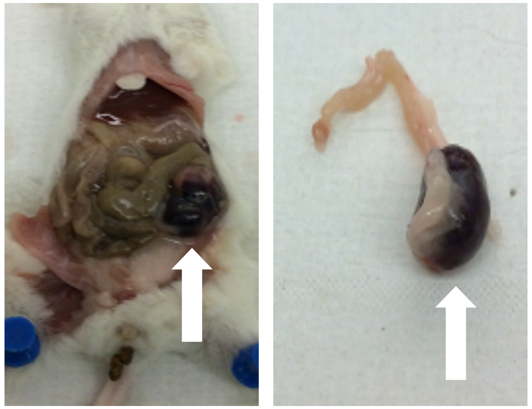

Figure S4

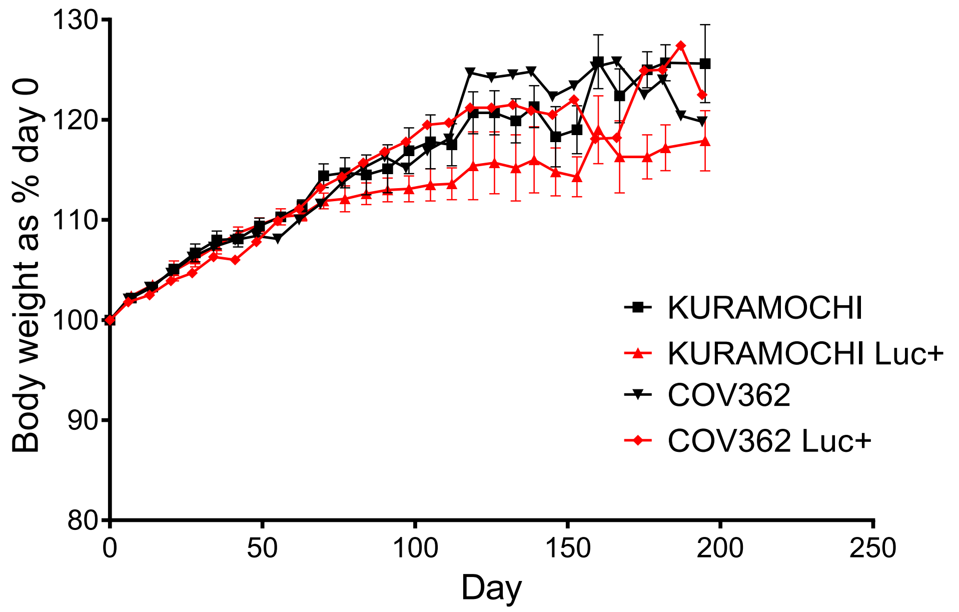

130
120
110
100
90
80
Body weight as % day 0
0
50
100
150
200
250
Day
KURAMOCHI
KURAMOCHI Luc+
COV362
COV362 Luc+

Figure S5

A. Ovarian non-HGSC cells

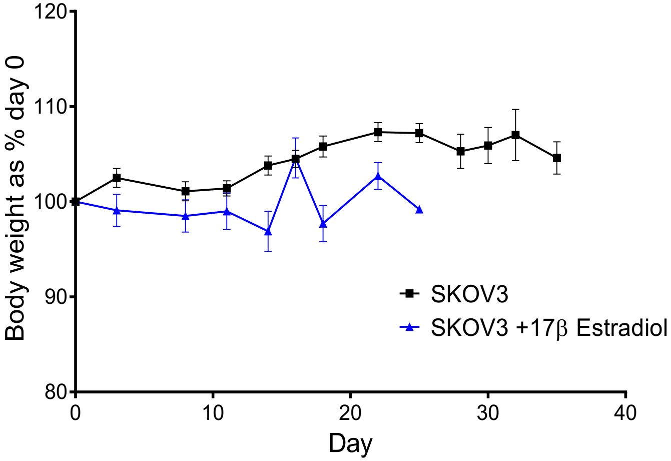

B. MCF7 – ERα⁺ control cell line

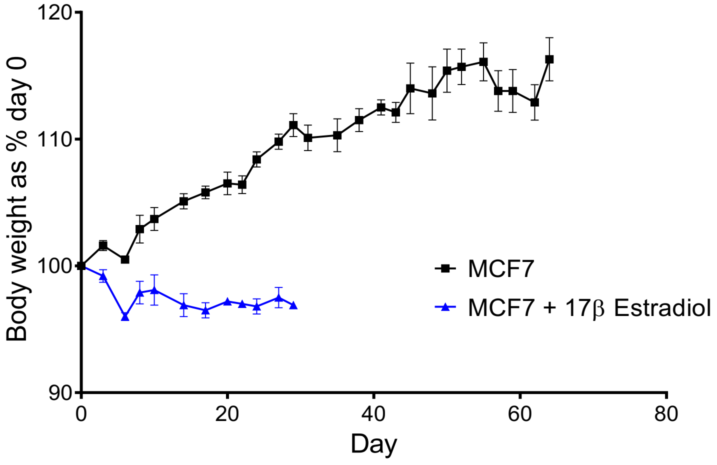

C. Ovarian HGSC cells

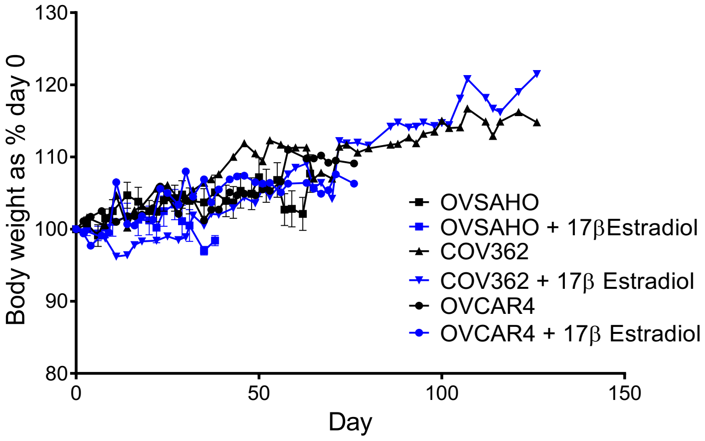

Figure S6

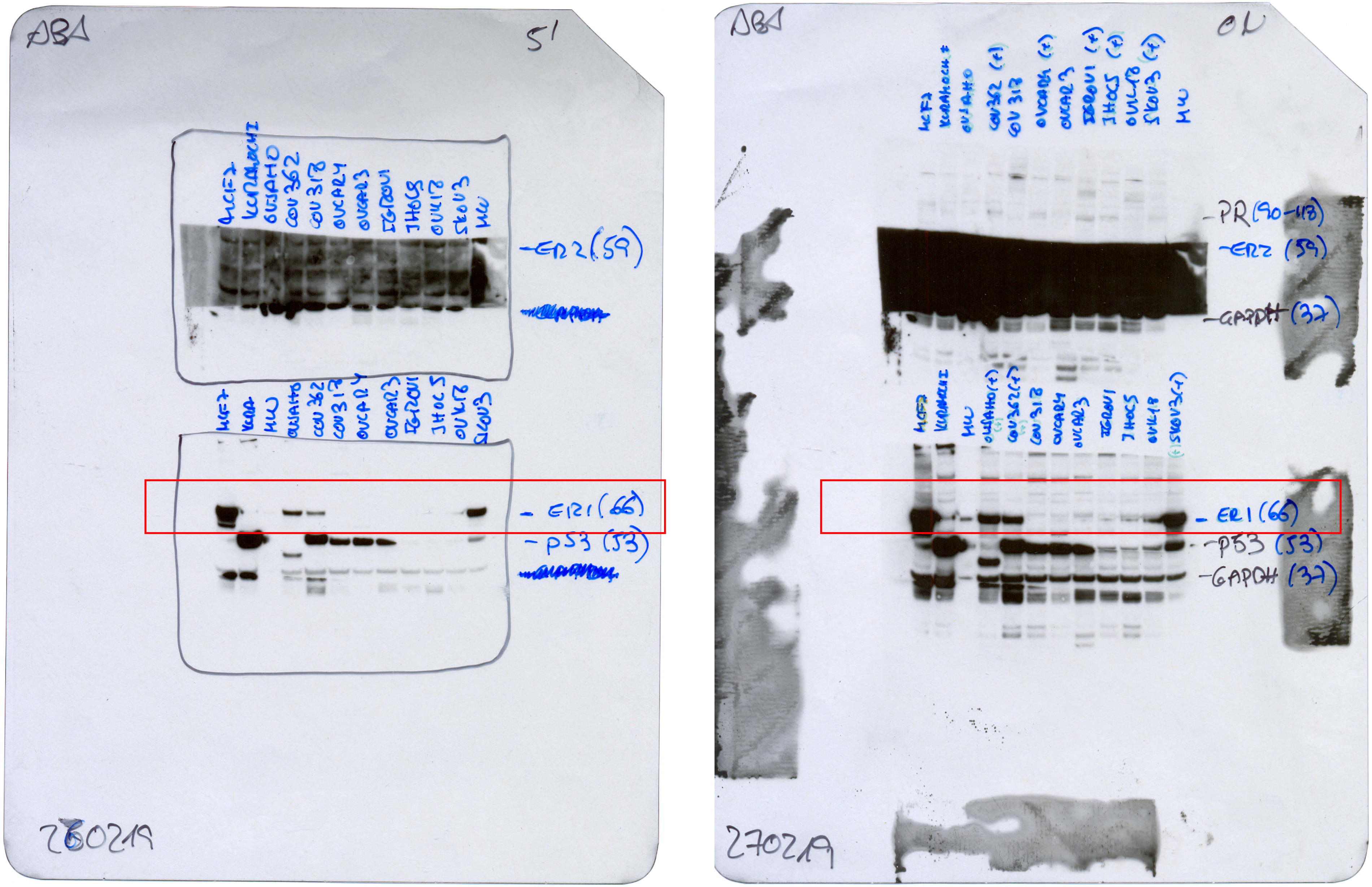

Figure S7
