# Supplementary material for: Identification of ovarian high-grade serous carcinoma cell lines that show estrogen-sensitive growth as xenografts in immunocompromised mice
Source: Sci Rep. 2020 Jul 1;10:10799. doi: 10.1038/s41598-020-67533-1 (PMC7329846; doi:10.1038/s41598-020-67533-1)
Supplement: Supplementary file 2 — Supplementary Information 2 [file 41598_2020_67533_MOESM2_ESM.docx]

**Supplementary figure legends**

**Figure S1.** Body weights, expressed as a percentage of body weight at day 0, of NSG mice growing subcutaneous xenografts of ovarian cancer. (A) Ovarian CCC cells. (B) Ovarian EAC cells. (C) and (D) Ovarian HGSC cells. *Abbreviations:* Luc^+^, luciferase-expressing cells.

**Figure S2.** Representative images of ovarian cancer cell line-derived tumors growing in the peritoneal cavity of NSG mice. (A) and (B) Ovarian CCC cells. (C) and (D) Ovarian EAC cells. (E) and (F) Ovarian HGSC cells. White arrows indicate the visible anatomical position of the tumors: (A, left and right) SKOV3 Luc^+^ tumors in the omentum; (B, left and right) JHOC5 tumors on the liver; (C, left and rights) OVK18 tumors in the omentum and unattached but near the liver and ovaries; (D, left and right) IGROV1 tumors in the omentum; (E) OVCAR4 tumors in the omentum (left) and isolated tumor (right); (F) a sheet of small COV318 Luc^+^ tumors on the liver (left) and the isolated liver with the tumors (right). *Abbreviations:* Luc^+^, luciferase-expressing cells.

**Figure S3.** Body weights, expressed as a percentage of body weight at day 0, of NSG mice growing intraperitoneal xenografts of ovarian cancer. (A) Ovarian CCC cells. (B) Ovarian EAC cells. (C) and (D) Ovarian HGSC cells. *Abbreviations:* Luc^+^, luciferase-expressing cells.

**Figure S4.** Representative images of HGSC cell line-derived tumors growing in the ovarian bursa of NSG mice. White arrows indicate the visible anatomical position of the tumors: (A) COV362, ovary was surrounded by a fluid-filled sack (3 individual mice); (B) COV362 Luc^+^, one ovary contains a massive tumor (left) and isolated ovary (containing the tumor), oviduct, and uterus (right); (C) KURAMOCHI, one ovary contains a massive tumor (left) and isolated ovary (containing the tumor), oviduct, and uterus (right). *Abbreviations:* Luc^+^, luciferase-expressing cells.

**Figure S5.** Body weights, expressed as a percentage of body weight at day 0, of NSG mice growing intrabursal xenografts of ovarian cancer. *Abbreviations:* Luc^+^, luciferase-expressing cells.

**Figure S6.** Body weights, expressed as a percentage of body weight at day 0, of NSG mice (with and without 17β-Estradiol supplementation) growing subcutaneous xenografts of ovarian cancer. *Abbreviations:* Luc^+^, luciferase-expressing cells.

**Figure S7.** Original and uncropped images of Western blots shown in Figure 4A.
